# Supplementary material for: Multi‐dataset identification of innovative feature genes and molecular mechanisms in keratoconus
Source: J Cell Mol Med. 2024 Sep 19;28(18):e70079. doi: 10.1111/jcmm.70079 (PMC11412914; doi:10.1111/jcmm.70079)
Supplement: Supplementary file 2 — Material S2. [file JCMM-28-e70079-s002.zip › 7.GSEA analysis/1.ARL11/KEGG_LEUKOCYTE_TRANSENDOTHELIAL_MIGRATION.html]

Details for gene set KEGG\_LEUKOCYTE\_TRANSENDOTHELIAL\_MIGRATION[GSEA]

|  || Dataset | ARL11.group.cls#h\_versus\_l |
| Phenotype | group.cls#h\_versus\_l |
| Upregulated in class | h |
| GeneSet | KEGG\_LEUKOCYTE\_TRANSENDOTHELIAL\_MIGRATION |
| Enrichment Score (ES) | 0.22582953 |
| Normalized Enrichment Score (NES) | 0.8543521 |
| Nominal p-value | 0.77120316 |
| FDR q-value | 1.0 |
| FWER p-Value | 1.0 |
Table: GSEA Results Summary

  

Fig 1: Enrichment plot: KEGG\_LEUKOCYTE\_TRANSENDOTHELIAL\_MIGRATION      
 Profile of the Running ES Score & Positions of GeneSet Members on the Rank Ordered List

  

| SYMBOL | TITLE | RANK IN GENE LIST | RANK METRIC SCORE | RUNNING ES | CORE ENRICHMENT || 1 | ITGA4 | na | 272 | 0.550 | 0.0310 | Yes |
| 2 | NOX3 | na | 549 | 0.466 | 0.0560 | Yes |
| 3 | CLDN5 | na | 852 | 0.409 | 0.0761 | Yes |
| 4 | RAPGEF4 | na | 1109 | 0.375 | 0.0952 | Yes |
| 5 | TXK | na | 1276 | 0.358 | 0.1157 | Yes |
| 6 | ITGAL | na | 2048 | 0.293 | 0.1142 | Yes |
| 7 | PIK3R3 | na | 2180 | 0.284 | 0.1304 | Yes |
| 8 | NOX1 | na | 2219 | 0.281 | 0.1491 | Yes |
| 9 | PIK3CG | na | 2870 | 0.247 | 0.1478 | Yes |
| 10 | ITGB2 | na | 4046 | 0.199 | 0.1279 | Yes |
| 11 | CLDN15 | na | 4053 | 0.199 | 0.1418 | Yes |
| 12 | PLCG1 | na | 4334 | 0.189 | 0.1471 | Yes |
| 13 | CLDN10 | na | 4680 | 0.176 | 0.1495 | Yes |
| 14 | ITK | na | 4854 | 0.170 | 0.1566 | Yes |
| 15 | PECAM1 | na | 5068 | 0.163 | 0.1620 | Yes |
| 16 | CLDN18 | na | 5112 | 0.162 | 0.1722 | Yes |
| 17 | CLDN9 | na | 5240 | 0.159 | 0.1797 | Yes |
| 18 | NCF4 | na | 5605 | 0.148 | 0.1797 | Yes |
| 19 | CLDN22 | na | 5685 | 0.145 | 0.1877 | Yes |
| 20 | NCF2 | na | 6048 | 0.136 | 0.1868 | Yes |
| 21 | PIK3R1 | na | 6079 | 0.135 | 0.1955 | Yes |
| 22 | PLCG2 | na | 6096 | 0.135 | 0.2046 | Yes |
| 23 | CLDN20 | na | 6192 | 0.132 | 0.2112 | Yes |
| 24 | VCAM1 | na | 6487 | 0.124 | 0.2115 | Yes |
| 25 | CLDN3 | na | 6601 | 0.122 | 0.2168 | Yes |
| 26 | CLDN23 | na | 6669 | 0.120 | 0.2233 | Yes |
| 27 | CXCR4 | na | 7115 | 0.109 | 0.2182 | Yes |
| 28 | CLDN19 | na | 7118 | 0.109 | 0.2258 | Yes |
| 29 | GNAI3 | na | 7891 | 0.095 | 0.2102 | No |
| 30 | VAV2 | na | 8664 | 0.080 | 0.1936 | No |
| 31 | PIK3R2 | na | 8783 | 0.078 | 0.1957 | No |
| 32 | CYBA | na | 8885 | 0.077 | 0.1982 | No |
| 33 | ESAM | na | 8917 | 0.076 | 0.2027 | No |
| 34 | MYL5 | na | 9267 | 0.071 | 0.1976 | No |
| 35 | ROCK1 | na | 9633 | 0.065 | 0.1917 | No |
| 36 | VAV3 | na | 9977 | 0.060 | 0.1860 | No |
| 37 | PIK3R5 | na | 10118 | 0.059 | 0.1861 | No |
| 38 | CLDN7 | na | 10343 | 0.056 | 0.1836 | No |
| 39 | PRKCA | na | 10617 | 0.052 | 0.1794 | No |
| 40 | BCAR1 | na | 10697 | 0.051 | 0.1807 | No |
| 41 | CTNND1 | na | 11094 | 0.047 | 0.1726 | No |
| 42 | MAPK11 | na | 11276 | 0.044 | 0.1705 | No |
| 43 | ACTB | na | 12961 | 0.027 | 0.1238 | No |
| 44 | GNAI2 | na | 13045 | 0.027 | 0.1233 | No |
| 45 | MAPK12 | na | 13078 | 0.026 | 0.1242 | No |
| 46 | RAPGEF3 | na | 13178 | 0.025 | 0.1231 | No |
| 47 | ACTN4 | na | 13347 | 0.024 | 0.1200 | No |
| 48 | PIK3CA | na | 13512 | 0.023 | 0.1169 | No |
| 49 | ACTN1 | na | 13743 | 0.021 | 0.1117 | No |
| 50 | PTPN11 | na | 14607 | 0.014 | 0.0878 | No |
| 51 | CTNNB1 | na | 14653 | 0.014 | 0.0874 | No |
| 52 | CTNNA1 | na | 15057 | 0.011 | 0.0766 | No |
| 53 | ROCK2 | na | 15225 | 0.009 | 0.0724 | No |
| 54 | ITGAM | na | 15359 | 0.009 | 0.0692 | No |
| 55 | RASSF5 | na | 15399 | 0.008 | 0.0686 | No |
| 56 | CLDN4 | na | 15840 | 0.005 | 0.0563 | No |
| 57 | ARHGAP35 | na | 16151 | 0.003 | 0.0475 | No |
| 58 | MYL12B | na | 16348 | 0.001 | 0.0419 | No |
| 59 | RAC1 | na | 16390 | 0.001 | 0.0408 | No |
| 60 | VASP | na | 16922 | -0.003 | 0.0257 | No |
| 61 | CDH5 | na | 17241 | -0.005 | 0.0168 | No |
| 62 | PTK2B | na | 17352 | -0.006 | 0.0141 | No |
| 63 | PRKCG | na | 17545 | -0.007 | 0.0090 | No |
| 64 | MAPK14 | na | 17567 | -0.007 | 0.0089 | No |
| 65 | RAP1B | na | 17707 | -0.008 | 0.0055 | No |
| 66 | CDC42 | na | 18361 | -0.013 | -0.0125 | No |
| 67 | F11R | na | 18558 | -0.014 | -0.0171 | No |
| 68 | SIPA1 | na | 18741 | -0.015 | -0.0213 | No |
| 69 | RAC2 | na | 18868 | -0.016 | -0.0238 | No |
| 70 | RAP1A | na | 18999 | -0.017 | -0.0264 | No |
| 71 | CLDN8 | na | 19025 | -0.017 | -0.0259 | No |
| 72 | MYL12A | na | 19058 | -0.017 | -0.0256 | No |
| 73 | ACTN2 | na | 19694 | -0.022 | -0.0424 | No |
| 74 | OCLN | na | 19863 | -0.023 | -0.0457 | No |
| 75 | VAV1 | na | 20088 | -0.024 | -0.0504 | No |
| 76 | ACTG1 | na | 20219 | -0.025 | -0.0524 | No |
| 77 | VCL | na | 20263 | -0.026 | -0.0518 | No |
| 78 | CD99 | na | 20895 | -0.030 | -0.0679 | No |
| 79 | MAPK13 | na | 20970 | -0.031 | -0.0678 | No |
| 80 | RHOA | na | 21886 | -0.038 | -0.0916 | No |
| 81 | RHOH | na | 22129 | -0.041 | -0.0956 | No |
| 82 | PTK2 | na | 22303 | -0.043 | -0.0976 | No |
| 83 | NCF1 | na | 23159 | -0.052 | -0.1186 | No |
| 84 | ARHGAP5 | na | 23754 | -0.059 | -0.1316 | No |
| 85 | PIK3CB | na | 23818 | -0.060 | -0.1292 | No |
| 86 | CLDN16 | na | 24265 | -0.066 | -0.1374 | No |
| 87 | CLDN6 | na | 24385 | -0.068 | -0.1361 | No |
| 88 | MYL9 | na | 24535 | -0.070 | -0.1355 | No |
| 89 | PIK3CD | na | 25759 | -0.087 | -0.1647 | No |
| 90 | CLDN1 | na | 26061 | -0.091 | -0.1669 | No |
| 91 | PXN | na | 26293 | -0.095 | -0.1669 | No |
| 92 | EZR | na | 26428 | -0.097 | -0.1639 | No |
| 93 | CTNNA2 | na | 26542 | -0.099 | -0.1601 | No |
| 94 | GNAI1 | na | 27299 | -0.115 | -0.1739 | No |
| 95 | MSN | na | 27836 | -0.127 | -0.1804 | No |
| 96 | THY1 | na | 27974 | -0.130 | -0.1752 | No |
| 97 | ITGB1 | na | 28114 | -0.134 | -0.1697 | No |
| 98 | CYBB | na | 28589 | -0.146 | -0.1731 | No |
| 99 | MYL7 | na | 29740 | -0.180 | -0.1936 | No |
| 100 | ICAM1 | na | 29811 | -0.182 | -0.1828 | No |
| 101 | MMP2 | na | 30057 | -0.190 | -0.1765 | No |
| 102 | CXCL12 | na | 30202 | -0.194 | -0.1669 | No |
| 103 | MYL2 | na | 30743 | -0.212 | -0.1675 | No |
| 104 | CLDN11 | na | 31438 | -0.237 | -0.1708 | No |
| 105 | CLDN14 | na | 32448 | -0.283 | -0.1799 | No |
| 106 | CTNNA3 | na | 32684 | -0.300 | -0.1656 | No |
| 107 | CLDN17 | na | 33095 | -0.329 | -0.1542 | No |
| 108 | JAM2 | na | 33406 | -0.352 | -0.1383 | No |
| 109 | CLDN2 | na | 33555 | -0.365 | -0.1168 | No |
| 110 | PRKCB | na | 33863 | -0.399 | -0.0975 | No |
| 111 | MMP9 | na | 33942 | -0.411 | -0.0707 | No |
| 112 | MYL10 | na | 34046 | -0.426 | -0.0436 | No |
| 113 | JAM3 | na | 34122 | -0.440 | -0.0147 | No |
| 114 | ACTN3 | na | 34217 | -0.455 | 0.0148 | No |
Table: GSEA details [plain text format]

  

Fig 2: KEGG\_LEUKOCYTE\_TRANSENDOTHELIAL\_MIGRATION      
 Blue-Pink O' Gram in the Space of the Analyzed GeneSet

  

Fig 3: KEGG\_LEUKOCYTE\_TRANSENDOTHELIAL\_MIGRATION: Random ES distribution      
 Gene set null distribution of ES for **KEGG\_LEUKOCYTE\_TRANSENDOTHELIAL\_MIGRATION**
